# Supplementary figures and images for: Treponema pallidum genome sequencing from six continents reveals variability in vaccine candidate genes and dominance of Nichols clade strains in Madagascar
Source: PLoS Negl Trop Dis. 2021 Dec 22;15(12):e0010063. doi: 10.1371/journal.pntd.0010063 (PMC8735616; doi:10.1371/journal.pntd.0010063)

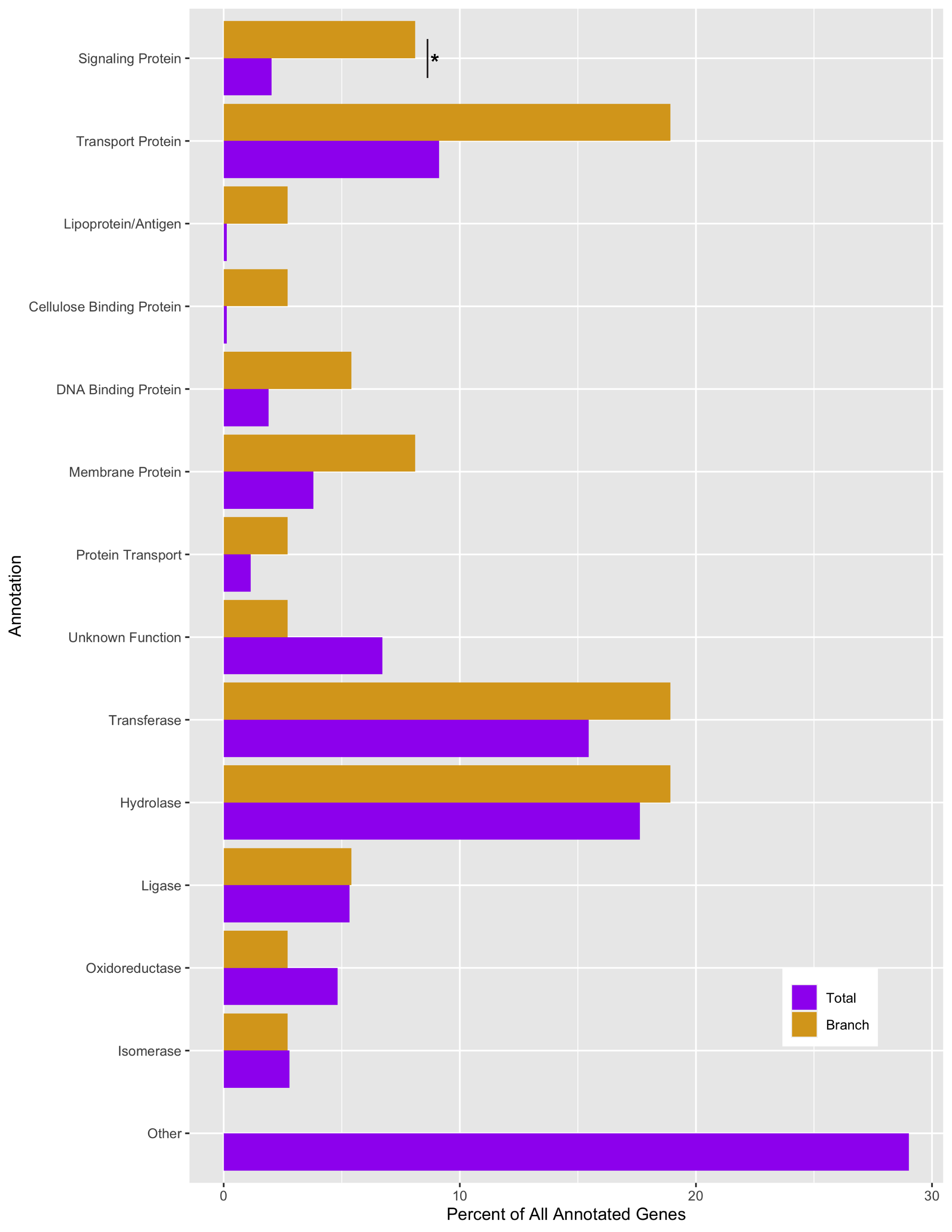

Supplement: S1 Fig — Percent of proteins in each category different between the SS14 ancestral clade node (N101) and the Nichols ancestral node (N001) (orange) were compared to annotations across the whole genome (purple). Overrepresentation was tested by Fisher’s exact test, *p < 0.05. (TIF) [file pntd.0010063.s001.tif]

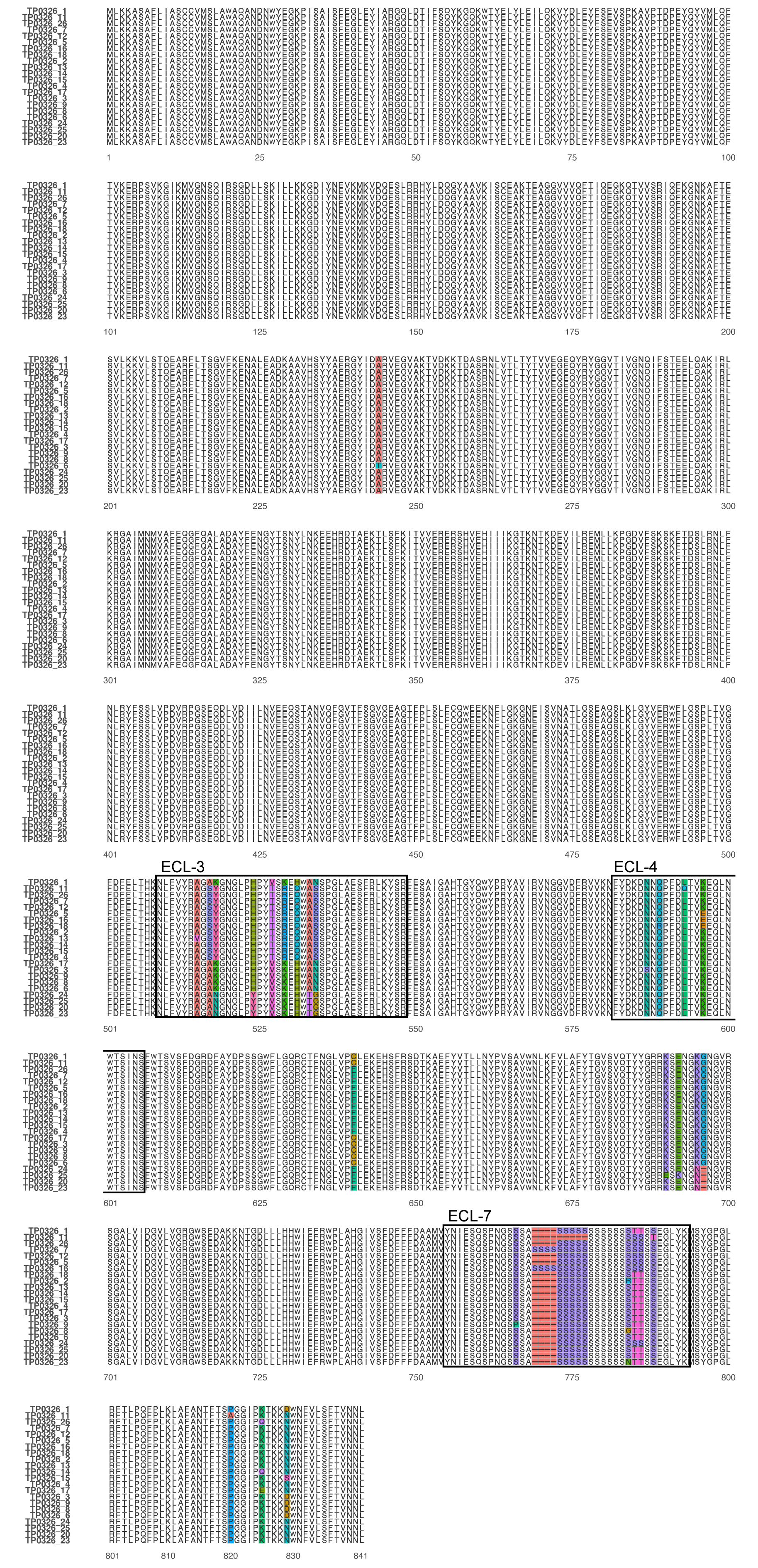

Supplement: S2 Fig — Multiple sequence alignment for all amino acid sequence variants. Polymorphic residues are highlighted, and positions of extracellular loops 3, 4, and 7 are shown. (TIF) [file pntd.0010063.s002.tif]

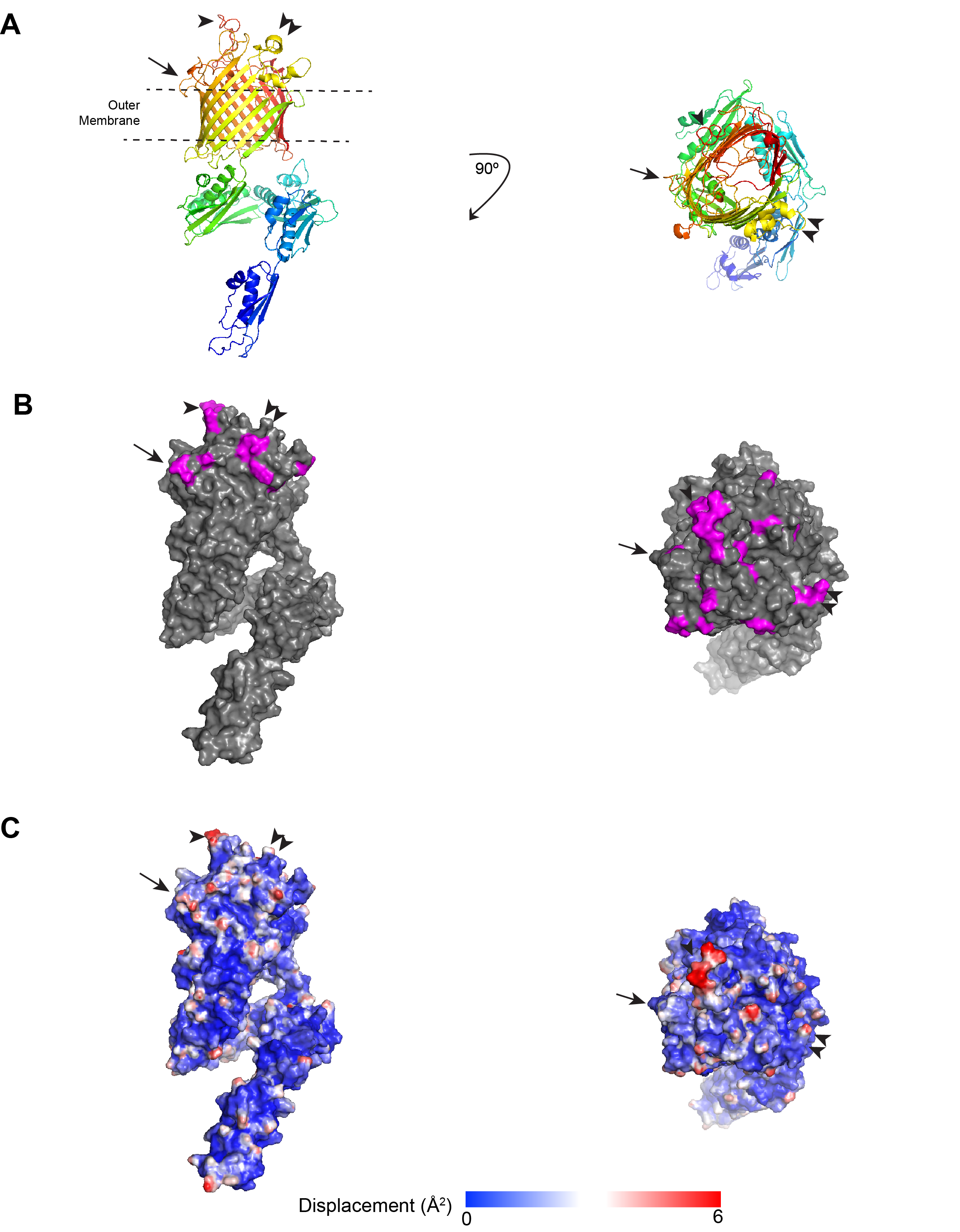

Supplement: S3 Fig — A) Side (left) and top (right) cartoon representation of TP0326, with a color gradient between blue at the N-terminus to red at the C-terminus. B) Side (left) and top (right) space-filling representation of TP0326, with polymorphic residue positions colored magenta. C) Side (left) and top (right) space-filling representation of TP0326, with atoms colored by average per atom displacement in all variants relative to the SS14 reference sequence. Arrows, single arrowheads, and double arrowheads point to positions of ECLs 4, 7, and 3, respectively. (TIFF) [file pntd.0010063.s003.tiff]

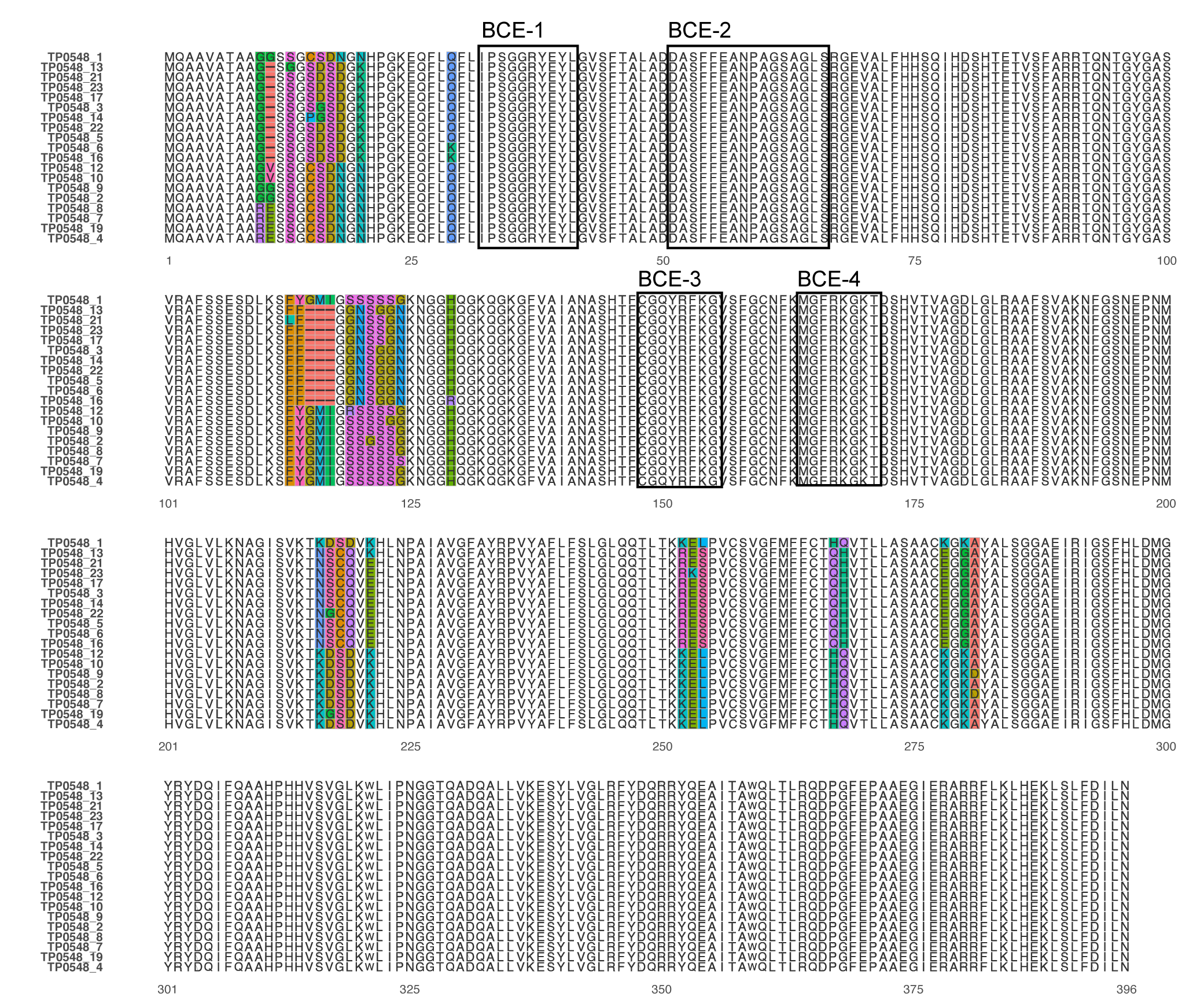

Supplement: S4 Fig — Multiple sequence alignment for all amino acid sequence variants. Polymorphic residues are highlighted, and positions of relevant predicted B cell epitopes are shown. (TIF) [file pntd.0010063.s004.tif]

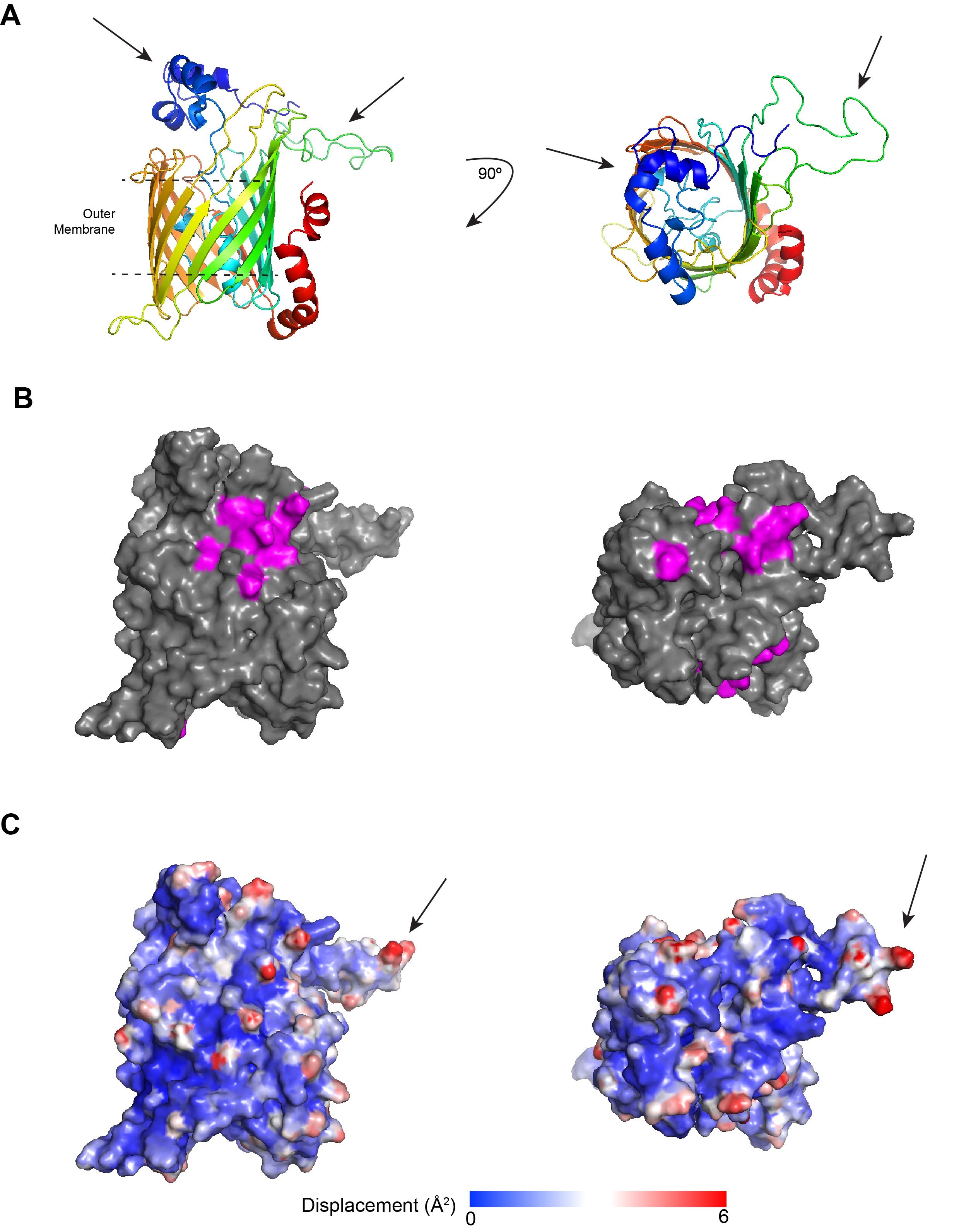

Supplement: S5 Fig — A) Side (left) and top (right) cartoon representation of TP0548, with a color gradient between blue at the N-terminus to red at the C-terminus. Arrows point to the flexible loops that contain predicted linear BCEs. B) Side (left) and top (right) space-filling representation of TP0548, with polymorphic residue positions colored magenta. C) Side (left) and top (right) space-filling representation of TP0966, with atoms colored by average per atom displacement in all variants relative to the SS14 reference sequence. Arrow points to ECL-2, which contains two invariant predicted BCEs. (TIFF) [file pntd.0010063.s005.tiff]

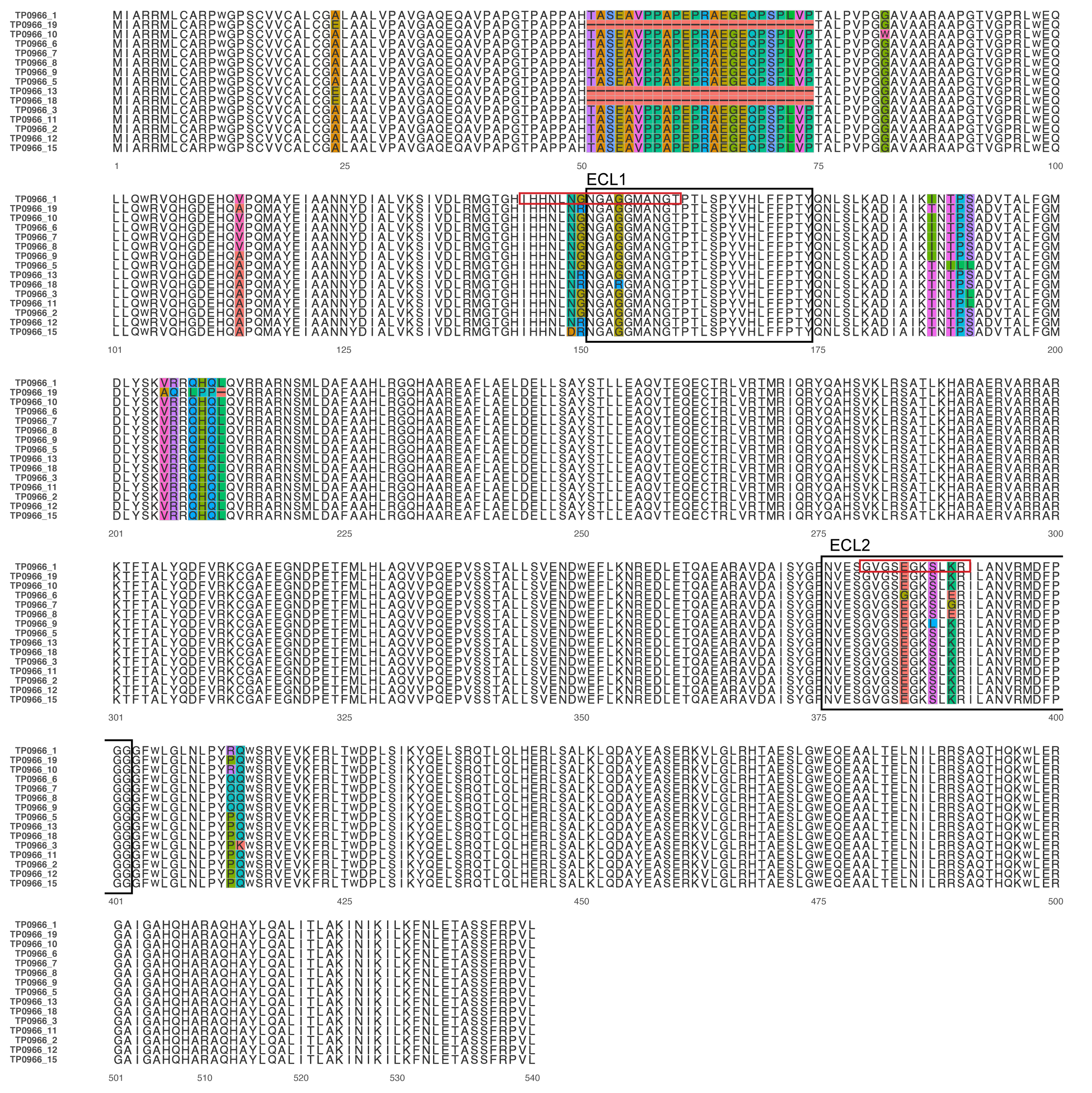

Supplement: S6 Fig — Multiple sequence alignment for all amino acid sequence variants. Polymorphic residues are highlighted. ECLs 1 and 2 are boxed, and the linear BCEs contained in the SS14 variant (#1) are marked in red. (TIF) [file pntd.0010063.s006.tif]

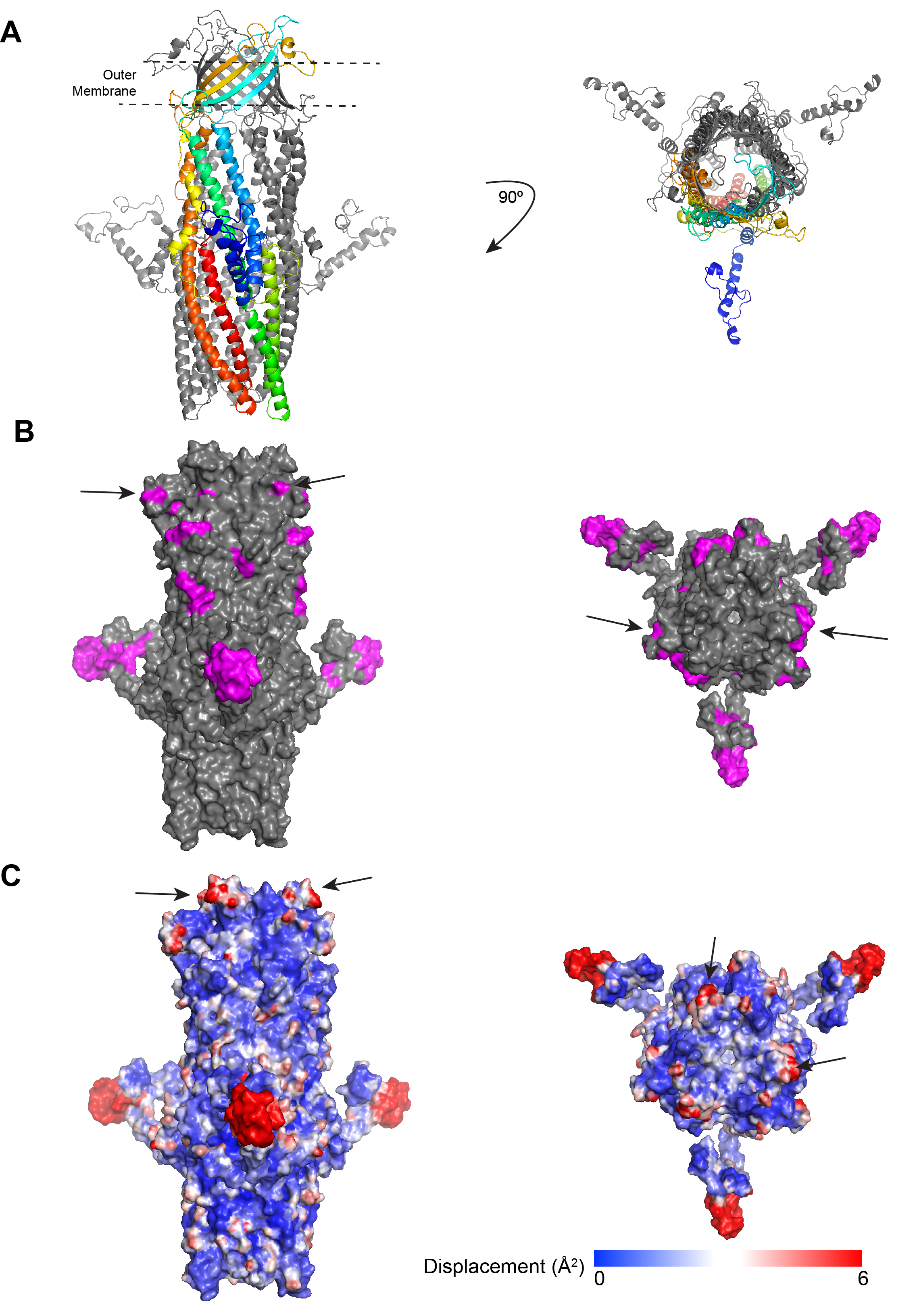

Supplement: S7 Fig — A) Side (left) and top (right) cartoon representation of TP0966, with a color gradient between blue at the N-terminus to red at the C-terminus. B) Side (left) and top (right) space-filling representation of TP0966, with polymorphic residue positions colored magenta. Arrow points to polymorphic residues in surface loops. C) Side (left) and top (right) space-filling representation of TP0966, with atoms colored by average per atom displacement in all variants relative to the SS14 reference sequence. Arrows point to the high displacement, non-polymorphic residues. (TIFF) [file pntd.0010063.s007.tiff]

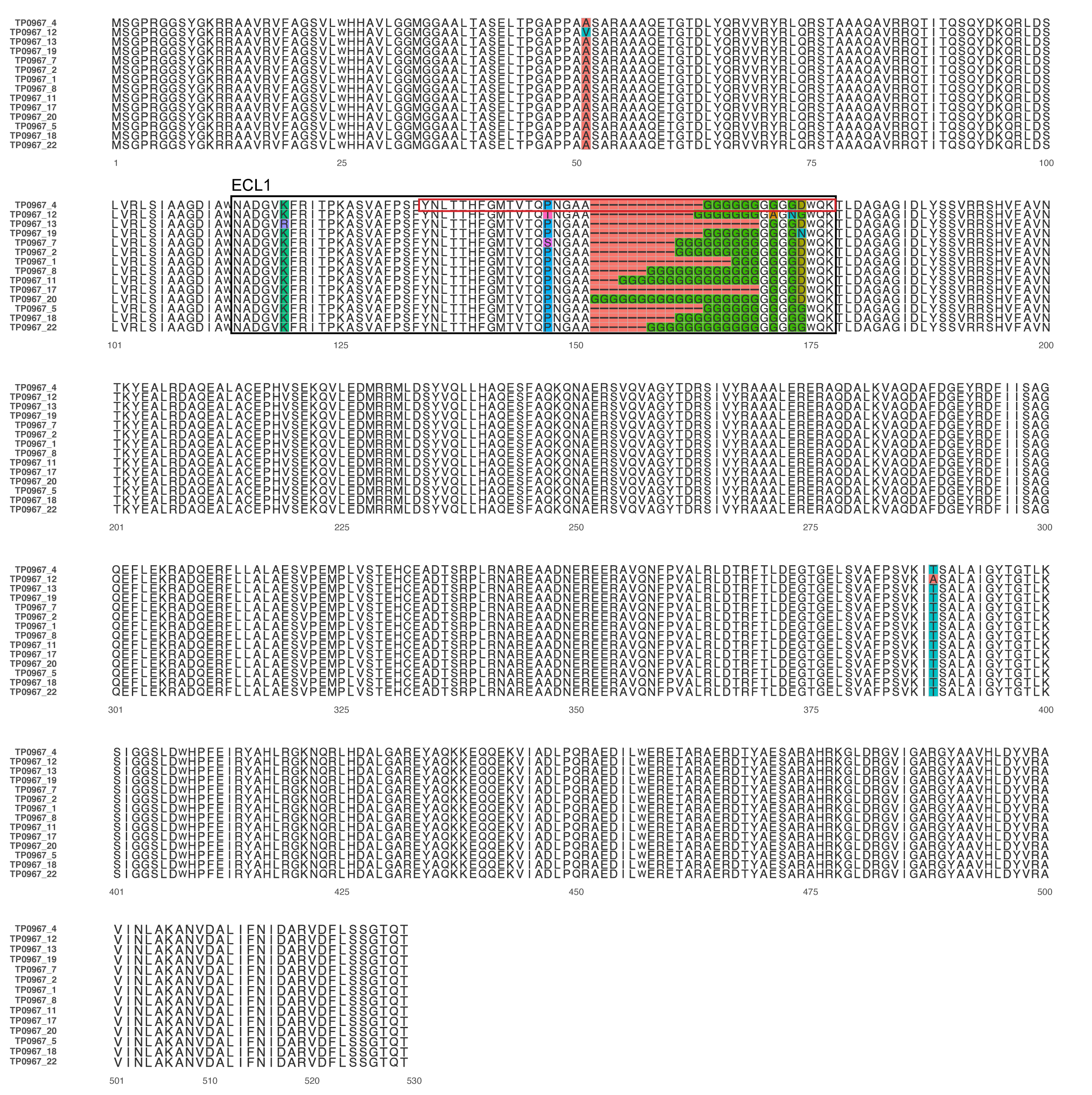

Supplement: S8 Fig — Multiple sequence alignment for all amino acid sequence variants. Polymorphic residues are highlighted. ECL1 is boxed, and the linear BCE contained in the SS14 variant (#4) is marked in red. (TIF) [file pntd.0010063.s008.tif]

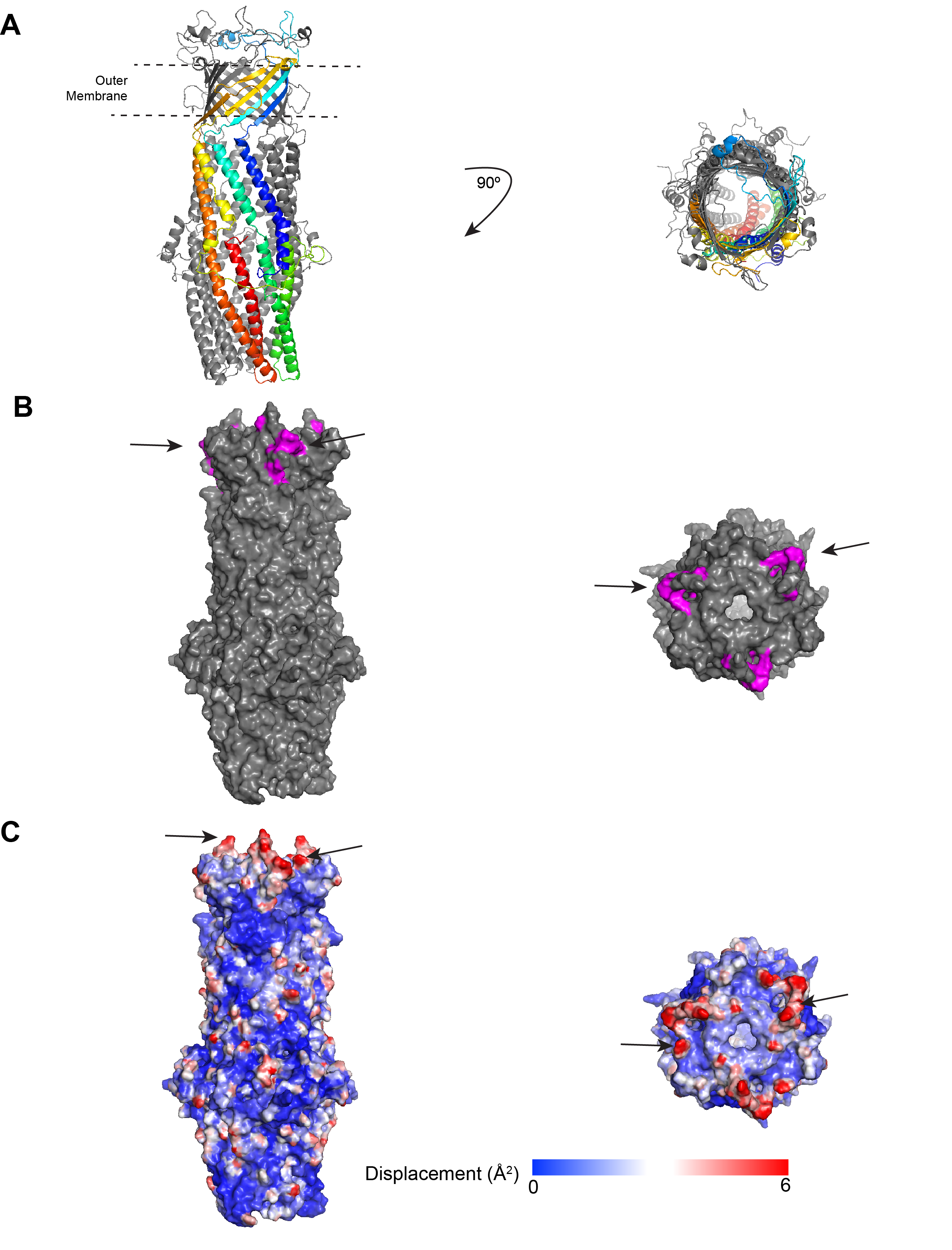

Supplement: S9 Fig — A) Side (left) and top (right) cartoon representation of TP0967, with a color gradient between blue at the N-terminus to red at the C-terminus. B) Side (left) and top (right) space-filling representation of TP0967, with polymorphic residue positions colored magenta. Arrow points to polymorphic residues in surface loops. C) Side (left) and top (right) space-filling representation of TP0967, with atoms colored by average per atom displacement in all variants relative to the SS14 reference sequence. Arrows point to the high displacement, non-polymorphic residues. (TIFF) [file pntd.0010063.s009.tiff]

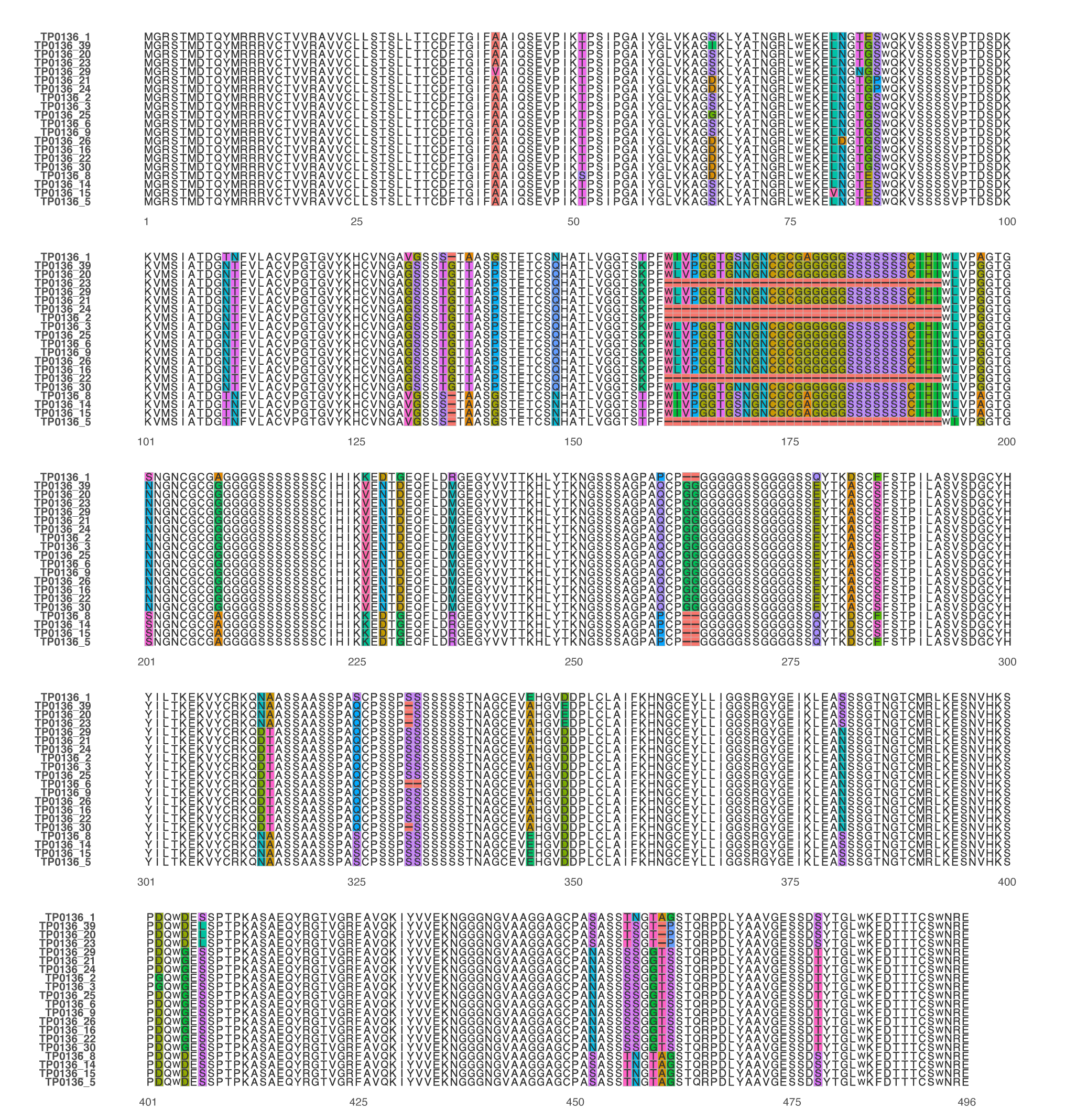

Supplement: S10 Fig — Multiple sequence alignment for all amino acid sequence variants. Polymorphic residues are highlighted. (TIF) [file pntd.0010063.s010.tif]

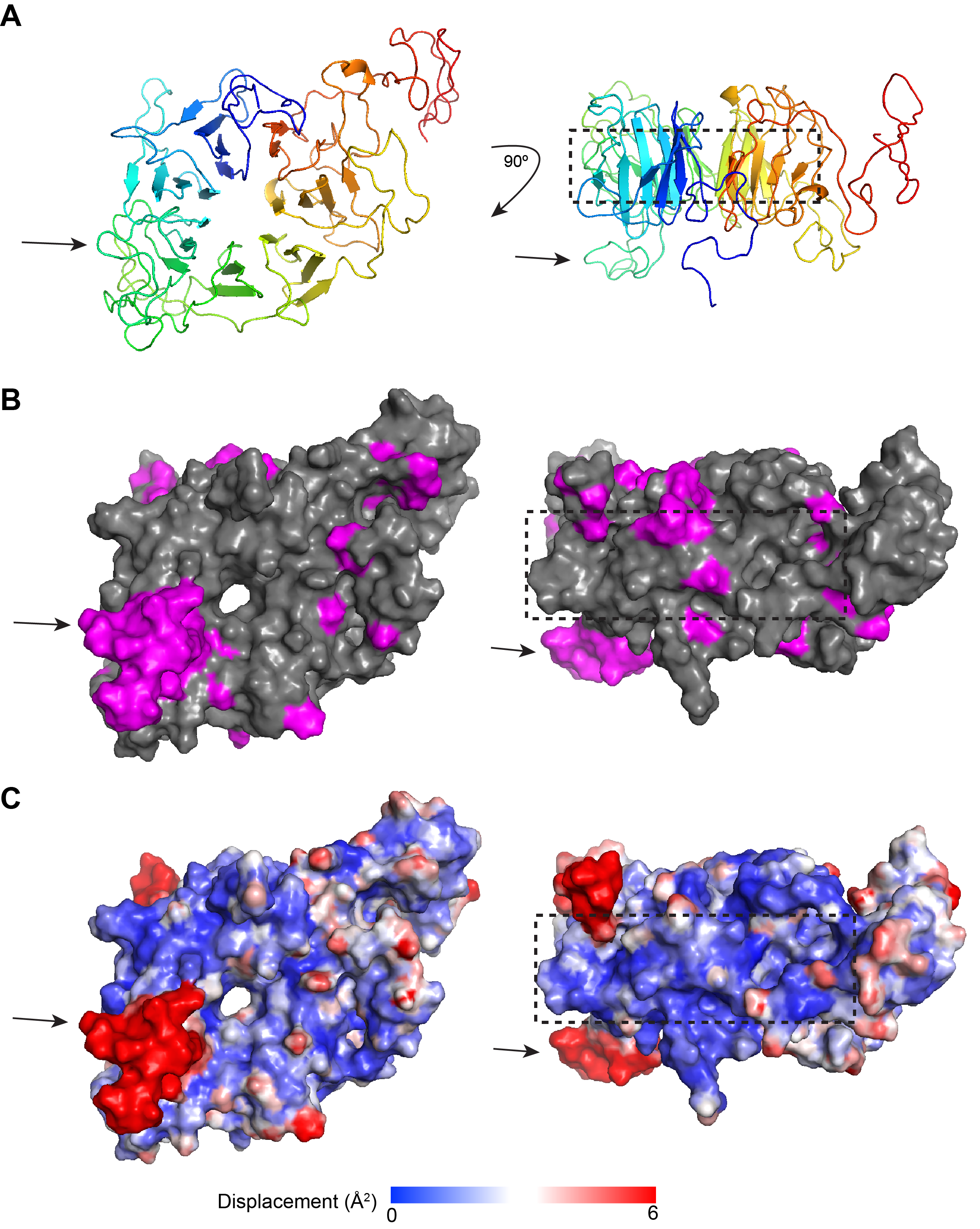

Supplement: S11 Fig — A) Side (left) and top (right) cartoon representation of TP0136, with a color gradient between blue at the N-terminus to red at the C-terminus. B) Side (left) and top (right) space-filling representation of TP0136, with polymorphic residue positions colored magenta. C) Side (left) and top (right) space-filling representation of TP0136, with atoms colored by average per atom displacement in all variants relative to the SS14 reference sequence. Boxed areas represent regions of low displacement in the β-strands. In all panels, arrows point to the large extracellular loop that is removed in variants found in several subclades. (TIFF) [file pntd.0010063.s011.tiff]
